# Minor Changes in the Hemagglutinin of Influenza A(H1N1)2009 Virus Alter Its Antigenic Properties

**DOI:** 10.1371/journal.pone.0025848

**Published:** 2011-10-11

**Authors:** Mari Strengell, Niina Ikonen, Thedi Ziegler, Ilkka Julkunen

**Affiliations:** Viral Infections Unit, Department of Vaccines and Immune Protection, National Institute for Health and Welfare, Helsinki, Finland; Duke-NUS Graduate Medical School, Singapore

## Abstract

**Background:**

The influenza A(H1N1)2009 virus has been the dominant type of influenza A virus in Finland during the 2009–2010 and 2010–2011 epidemic seasons. We analyzed the antigenic characteristics of several influenza A(H1N1)2009 viruses isolated during the two influenza seasons by analyzing the amino acid sequences of the hemagglutinin (HA), modeling the amino acid changes in the HA structure and measuring antibody responses induced by natural infection or influenza vaccination.

**Methods/Results:**

Based on the HA sequences of influenza A(H1N1)2009 viruses we selected 13 different strains for antigenic characterization. The analysis included the vaccine virus, A/California/07/2009 and multiple California-like isolates from 2009–2010 and 2010–2011 epidemic seasons. These viruses had two to five amino acid changes in their HA1 molecule. The mutation(s) were located in antigenic sites Sa, Ca1, Ca2 and Cb region. Analysis of the antibody levels by hemagglutination inhibition test (HI) indicated that vaccinated individuals and people who had experienced a natural influenza A(H1N1)2009 virus infection showed good immune responses against the vaccine virus and most of the wild-type viruses. However, one to two amino acid changes in the antigenic site Sa dramatically affected the ability of antibodies to recognize these viruses. In contrast, the tested viruses were indistinguishable in regard to antibody recognition by the sera from elderly individuals who had been exposed to the Spanish influenza or its descendant viruses during the early 20^th^ century.

**Conclusions:**

According to our results, one to two amino acid changes (N125D and/or N156K) in the major antigenic sites of the hemagglutinin of influenza A(H1N1)2009 virus may lead to significant reduction in the ability of patient and vaccine sera to recognize A(H1N1)2009 viruses.

## Introduction

During the recent two years, the pandemic influenza A virus of swine origin, influenza A(H1N1)2009 virus, has been the predominant circulating influenza virus in most parts of the world. The virus has infected millions of people and the infection has lead to the death of at least 18 400 individuals. In Finland the first cases of the influenza A(H1N1)2009 were identified in May 2009. During September the first local outbreaks occurred in garrisons and schools, after which the virus spread rapidly in the general population. The peak pandemic activity was observed during weeks 43–49 and by the end of the year the epidemic was over in Finland [1], [2]. During the 2010–2011 epidemic season influenza A(H1N1)2009 viruses were identified from the beginning of December 2010 until middle of March 2011.

In serosurveys elderly individuals were found to have pre-existing, cross-reactive antibodies against the novel 2009 pandemic virus that were likely originating from previous infections with antigenically related viruses such as the 1918 influenza virus and its immediate descendants that were circulating during the early decades of the 20^th^ century [3] [4]–[8]. Except for the elderly, large segments of the human population throughout the world lacked protective immunity against the novel influenza A(H1N1)2009 virus and were thus susceptible to the virus infection. Until now, likely due to limited immunological pressure in the general population, the virus has not yet undergone significant genetic or antigenic changes.

Through the hemagglutinin (HA) the influenza virus binds to sialic-acid receptors on the host cell surface, after which the virus is internalized and the viral genome enters the nucleus in order to initiate viral RNA synthesis. Since the HA is situated on the surface of the viral particles it is also a target for immune response, especially antibodies. The major antigenic epitopes in the HA molecule mutate frequently enabling the virus to escape immune responses. Recent reports on the evolution of influenza A(H1N1)2009 describe mutations S183P (amino acid numbering throughout the text starts from the mature HA0 without signal peptide) and I191L in HA that enhance viral replication in cell culture and in embryonated hens' eggs [9]. Other mutations, such as D127E, S183P and D222G have been shown to be associated with a more virulent phenotype in humans or mice [2], [10]–[12]. The D127E and S183P mutations have lead to antigenic changes and impaired recognition by ferret antisera raised against the A/California/07/2009 virus [11]. Recently, a new frequently observed mutation, E374K has been found [13]. This mutation locates in the HA oligomerization interface and is also part of a known antigenic site. This mutation is not unambiguously associated with severe disease, but interestingly it has been detected in pandemic vaccine breakthrough infections. These viruses typically had N125D substitution in their HA molecule [14]. During the year 2010 viruses with double mutations N125D and E374K have been found with increased frequency in the southern hemisphere [14]. These viruses have been associated with several vaccine breakthrough infections and were identified in a number of fatal cases [14]. The N125D mutation is located in the Sa epitope of A(H1N1)2009 HA [2] and in an avian influenza strain (A/Mallard/Pennsylvania/10218/84(H5N2)) the corresponding amino acid was reported to cause antigenic drift as an immune escape mutant [15]. However, antigenic analysis with ferret anti-sera showed no remarkable differences in antibody titers against the N125D virus as compared to the A/California/07/2009 vaccine virus [14].

Genetic characterization of the influenza A(H1N1)2009 viruses that circulated in Finland and elsewhere during 2009 show that these viruses were closely related to A/California/07/2009 vaccine virus [2], [6], [16]. At present, genetic analyses of the HA or neuraminidase (NA) genes do not reveal any changes that may have led to the selection of a virus with increased epidemic potential or exceptionally high virulence. However, the antigenic characteristics of these viruses have not been systematically analyzed in humans. Here we report that several amino acid changes, namely N125D, N156K, S162R, and E374K, are associated with reduced recognition by sera collected from patients having experienced natural A(H1N1)2009 infection or having received vaccination as analyzed by the hemagglutination inhibition (HI) test. Especially, double mutations N125D and N156K (2009 virus isolate) or N125D and E374K (2010 virus isolates) were associated with declined antibody recognition in vaccinated individuals.

## Results

### Analysis of amino acid sequences and prediction of the structure of HA of influenza A(H1N1)2009 virus strains

**Figure 1:**
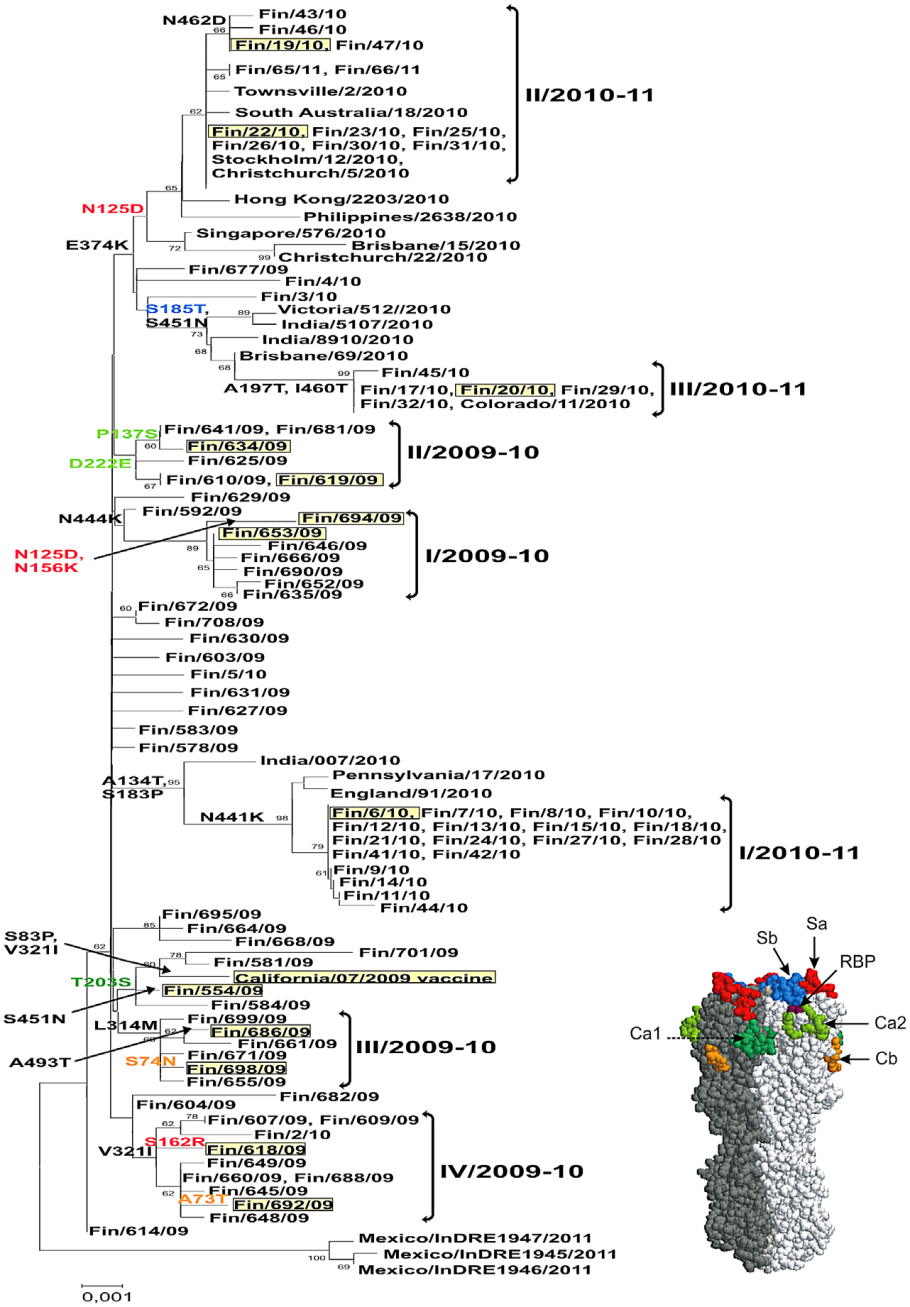
Phylogenetic tree of the HA of influenza A(H1N1)2009 strains. The strains included were identified in Finland during two epidemic seasons, 2009–10 and 2010–2011. Some representative viruses from other parts of the world are included for comparison. All sequences included in the phylogenetic tree constitute the entire 1698 nucleotide long coding region of HA. The horizontal lines are proportional to the number of nucleotide changes. The phylogenetic tree was constructed using the Neighbor-Joining method with Mega software version 4. Viruses used in HI tests are marked with yellow boxes. Signature amino acid changes are indicated with the colors which represent different antigenic epitopes of HA (Sa in red, Sb in blue, Ca1 in dark green, Ca2 in lighter green and Cb in orange). Amino acid changes marked in black do not locate in expected antigenic epitopes.

**Figure 2:**
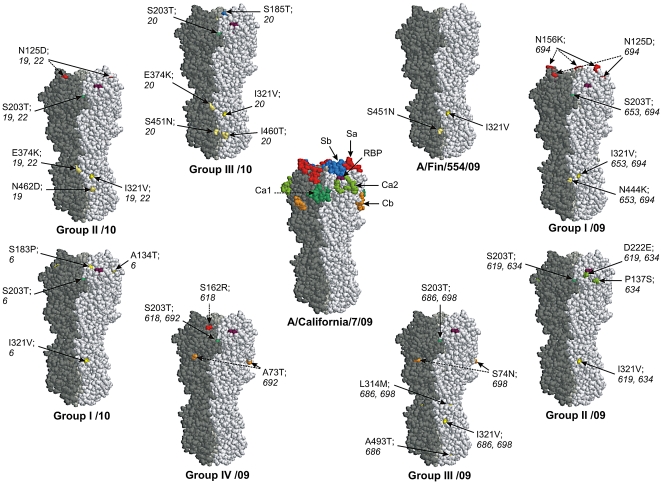
Schematic representation of amino acid differences in the HA molecule between the Finnish influenza A(H1N1)2009 viruses and the vaccine virus, A/California/07/2009. In the middle, a side view of the trimeric structure of HA molecule of influenza A(H1N1(2009) (A/California/04/2009; RCSB Protein Bank accession number 3LZG) with previously identified H1 protein-specific antigenic sites (Sa in red, Sb in blue, Ca1 in darker green, Ca2 in lighter green and Cb in orange) of influenza A(H1N1) viruses and with the receptor binding pocket (RBP, purple) is shown. Different monomers are shown in various shades of grey color. The amino acid changes of Finnish A(H1N1)2009 viruses compared to A/California/07/2009, the vaccine strain, are illustrated in the trimeric HA structure. Amino acid changes in the antigenic sites are colored as in A/California/07/2009 HA structure. Amino acid changes outside the antigenic sites are shown in yellow. Changes are illustrated by amino acid residue number and with serial number of virus where the respective amino acid change has been observed.

In order to get a better understanding of the antigenic evolution of recent pandemic A(H1N1)2009 viruses thorough characterization of the HA gene of influenza A(H1N1)2009 viruses identified in Finland during the epidemic seasons 2009–2010 and 2010–2011 were carried out. Based on the sequence analysis and comparisons of the HA molecules at the amino acid level the viruses identified in the 2009–2010 season clustered in four genetic groups I–IV/2009 [2] while the viruses identified during the 2010–2011 epidemic season formed three groups that are clearly district from each other and the 2009–2010 viruses (Figure 1). The viruses included in the phylogenetic tree shared no common source of transmission in regard to location of virus isolation or isolation date. A/Finland/554/2009 virus was one of the first influenza A(H1N1)2009 viruses isolated in Finland in June 2009 [17]. Its HA nucleotide sequence is almost identical with that of the A/California/07/2009 virus HA. The HA amino acid sequences of representative Finnish A(H1N1)2009 viruses identified during the last two epidemic seasons were compared with the vaccine strain, A/California/07/2009, and with the Spanish influenza virus, A/South Carolina/1/1918, a human seasonal A/H1N1 virus, A/Puerto Rico/8/1934, and the swine influenza virus, A/New Jersey/8/1976, which caused an epidemic in a garrison in Fort Dix in 1976. The amino acid alignment of HA showed that influenza A(H1N1)2009 viruses, including the vaccine strain, are very much alike (Figure S1). Compared to the vaccine strain, the Finnish A(H1N1)2009 viruses have two to five amino acid changes in their HA1 region, which are distributed to all the major antigenic epitopes. One conserved change in all Finnish A(H1N1)2009 virus strains, except A/Finland/554/2009, was S203T which is located in the antigenic epitope Ca1 of HA, close to the receptor binding pocket (Figure 2). This mutation has also been detected globally in majority of influenza A(H1N1)2009 viruses [18]. In addition to this common change, most of the Finnish A(H1N1)2009 viruses showed one or two additional amino acid changes in other antigenic epitopes. Of potential interest in respect to antibody recognition is the group I/2009–2010 virus, A/Finland/694/2009, which has N125D and N156K amino acid substitutions on the distal end of the HA molecule locating to the antigenic site Sa (Figure 2 and Figure S2). In addition, novel double mutations (N125D and E374K) were also found in the group II/2010–2011 viruses, namely in A/Finland/19/2010 and A/Finland/22/2010 viruses. The amino acid change E374K has been identified in some A(H1N1)2009 viruses isolated in Finland (Figure 1) and elsewhere [2], [13], [14]. Additional mutations at potential antigenic sites, in relation to the California vaccine virus, were also identified at positions 73, 74, 137, 162, 185, and 222 (Figure 2 and Figure S2).

### Finnish influenza A(H1N1)2009 virus strains show considerable antigenic heterogeneity

Sequence analysis of the HA gene revealed several amino acid changes that have occurred in the viruses that circulated in Finland during the 2009–2010 and 2010–2011 epidemic seasons. By analyzing the nucleotide sequence and the molecular structure of HA it is, however, difficult to estimate whether these changes have any importance in antibody recognition. To analyze the impact of amino acid changes on the antigenic characteristics of circulating epidemic viruses we selected two representative viruses from each of the four epidemic groups of the 2009–2010 viruses and one to two viruses from the three epidemic groups of the 2010–2011 viruses (Figure 1). We determined antibody levels against the thirteen Finnish influenza strains and the vaccine A/California/07/2009 virus by HI test in a set of 120 serum samples (Table 1). The 2010 (epidemic season 2010–2011) viruses were tested in separate sets of analyses with the same panel of sera and the data is therefore tabled separately (Table 1, upper and lower panel, respectively). The vaccine strain, A/California/07/2009, was used as a control in both analysis series and showed highly reproducible mean antibody levels (Table 1; GMTs 28.5 and 29.9). The geometric mean titers obtained by HI test using different virus isolates range from 14.7 to 38.9. The lowest antibody titers were detected against the viruses with amino acid changes in the Sa region of the HA e.g. A/Finland/694/2009, A/Finland/618/2009 (Table 1) and A/Finland/19/2010, A/Finland/22/2010 (Table 1). Some of the lowest antibody titers were also observed against A/Finland/698/2009, which had amino acid change in the antigenic epitope Cb (Figure 2 and Figure S2).

**Table 1:** The antibody geometric mean titers with 95% confidencial intervals (CI).

Virus strain	group	Amino acid changes*	Geometric mean (95%CI)
A/California/7/09 E2	California-like		28.5 (22.6–35.9)
A/California/7/09 M3	California-like		24.0 (19.4–29.7)
A/Finland/554/09	California-like	I321V, S451N	27.8 (22.9–33.7)
A/Finland/653/09	I/2009	S203T, I321V, N444K	31.5 (25.9–38.4)
A/Finland/694/09	I/2009	S203T,N125D,N156K, I321V, N444K	14.7 (12.8–16.8)
A/Finland/619/09	II/2009	S203T, D222E, I321V	33.9 (27.6–41.6)
A/Finland/634/09	II/2009	S203T,P137S, D222E, I321V	26.4 (21.3–32.6)
A/Finland/686/09	III/2009	S203T, I321V,L314MA493T	28.8 (23.6–35.2)
A/Finland/698/09	III/2009	S203T, S74N, I321V, L314	21.1 (17.8–25.1)
A/Finland/618/09	IV/2009	S203T, S162R	22.6 (18.7–27.4)
A/Finland/692/09	IV/2009	S203T, A73T	32.9 (26.9–40.2)

Virus strain	group	Amino acid changes*	Geometric mean (95% CI)
A/California/7/09 E2	California-like		29.9 (22.6–37.1)
A/Finland/6/10	I/2010	S203T, I321V,A134T,S183P	33.1 (26.1–40.5)
A/Finland/19/10	II/2010	S203T, N125D, I321V, E374K, N462D	28.0 (22.5–33.3)
A/Finland/22/10	II/2010	S203T, N125D, I321V, E374K	26.4 (21.2–31.5)
A/Finland/20/10	III/2010	S203T, S185T, I321V,E374K,S451N,I460T	38.9 (30.2–47.8)

The antibody titers of a set of 120 sera were analyzed against A/California/07/2009 virus and thirteen pandemic influenza A(H1N1) 2009 viruses isolated in Finland during 2009–2010 and 2010–2011 seasons. The group of each virus is determined by sequencing the hemagglutinin gene and it's location in the phylogenetic tree. *Amino acid changes, which locate in the surface of the HA molecule, as compared to A/California/7/09 virus.

### Antibody titers for cell culture-grown viruses correlate with egg-propagated virus in HI test

**Figure 3:**
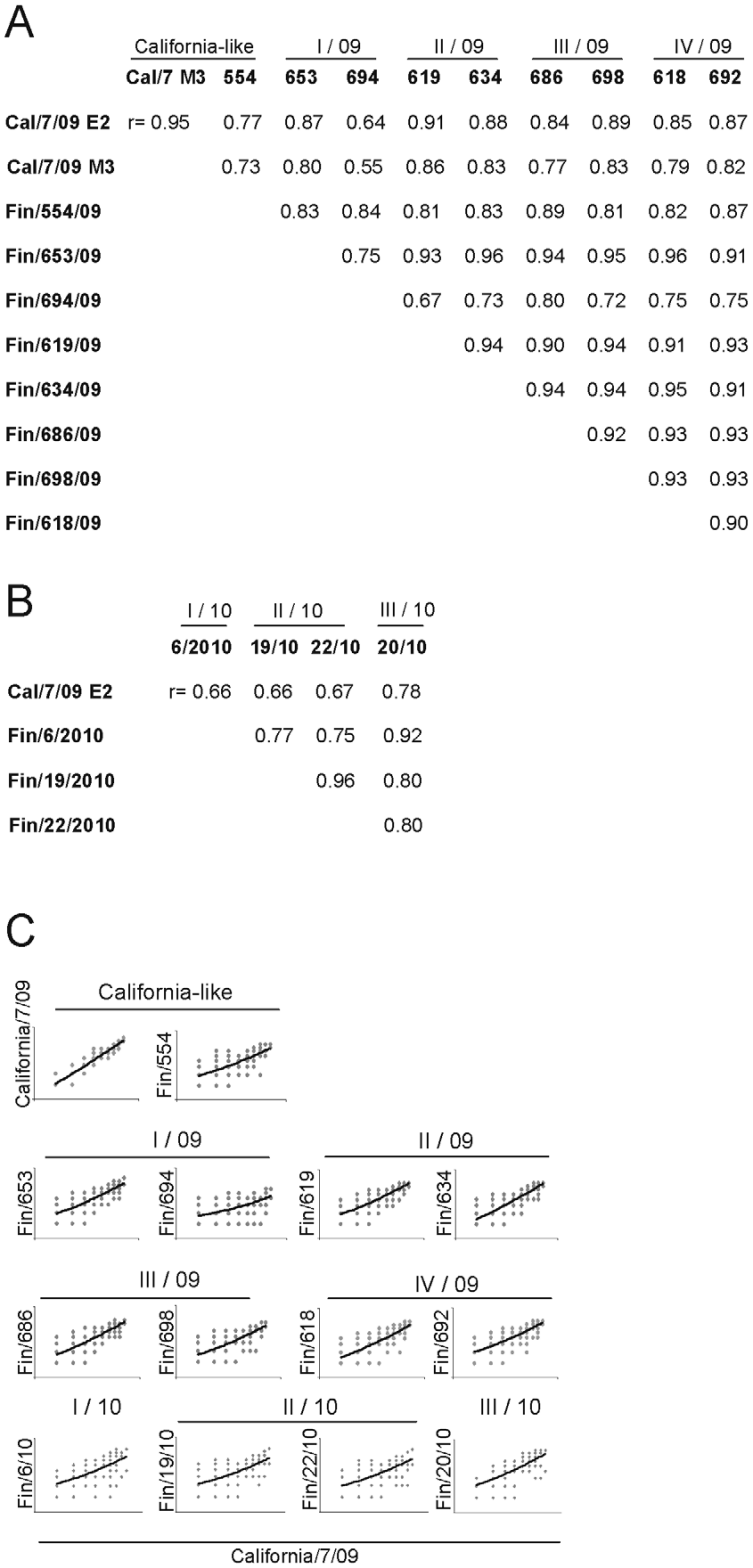
Correlation coefficiencies of the antibody titers between different virus isolates used in the HI test. The Pearson product moment correlation coefficients (r) were calculated for all pairs for 2009–10 viruses (panel A) and for 2010–11 viruses (panel B). All correlation coefficients were significant (p<0.01). C) The scatter plots and trend lines of antibody titers for each Finnish A(H1N1)2009 virus compared to the A/California/07/2009 vaccine virus.

All Finnish strains were isolated and propagated in Madin-Darby canine kidney (MDCK) cells. For comparison we tested both MDCK- and egg-grown vaccine strain, A/California/07/2009, in the HI test. The geometric mean titers to batches of MDCK- and egg-grown A/California/07/2009 were comparable although analyses with egg-grown virus stocks gave somewhat higher antibody levels (Table 1). The correlation coefficient for antibody titers obtained by HI test varied between 0.55 and 0.96, which indicated a strong positive correlation between the vaccine virus and Finnish epidemic strains (Figure 3A and B). Correlation coefficients for all tested viruses were significant, p<0.001, which suggested that MDCK- and egg-grown viruses were comparable in HI test. However, the scatter blots in Figure 3 C showed that the correlation between different viruses, especially Finland/694/2009, Finland/6/2010, Finland/19/2010, and Finland/22/2010, is better for sera with high antibody titers (>40) as compared to that observed with sera having lower titers, where the spots (HI values) seemed to be more dispersed.

### The antibodies induced by natural infection with influenza A(H1N1)2009 show differential recognition of viruses with mutations in antigenic epitopes

**Figure 4:**
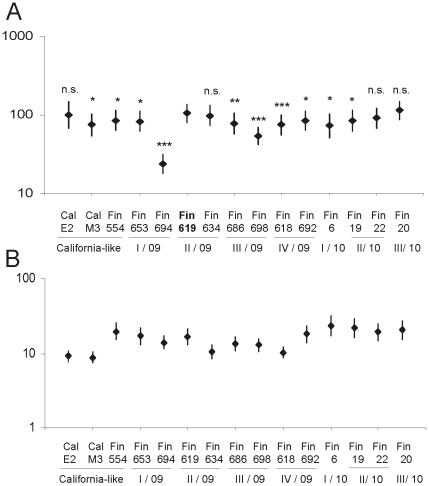
Geometric mean titers of anti-A(H1N1)2009 virus antibodies among military conscripts and the elderly. A) Serum samples from military conscripts (Dragsvik garrison) who had undergone a natural influenza A(H1N1)2009 virus infection (n = 40) were analyzed by HI test and geometric mean titers (+/− 95% confidence intervals, CI) for each virus were calculated. The significance of differences between A/Finland/619/09 and other viruses (California-like, viruses from groups I–IV/2009–10, and viruses from groups I–III/2010–11) were calculated using Student's paired t-test, * p<0.01, ** p<0.001, *** p<0.0001. The index virus, A/Finland/619/09 (bolded) was isolated from Dragsvik garrison during the outbreak in September 2009. B) The serum samples collected before pandemic in 2004–2005 from the elderly people (80 years of age or over) were analyzed by HI test and geometric mean titers (+/− 95% CI) for each virus were calculated.

**Figure 5:**
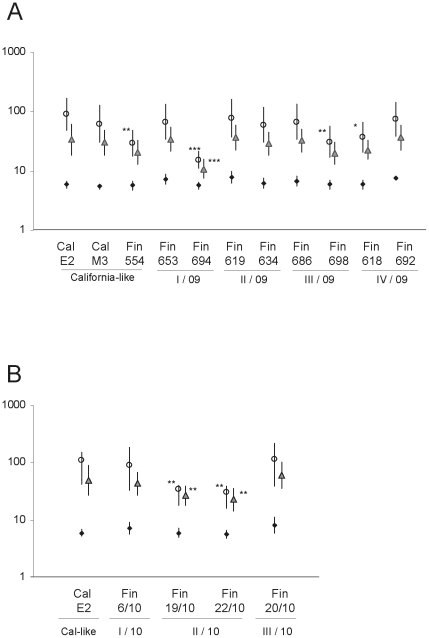
Geometric mean titers of anti-influenza A(H1N1)2009 virus antibodies among pandemic influenza-vaccinated individuals. The serum samples from persons vaccinated with one dose of pandemic influenza A(H1N1)2009 virus vaccine were collected on day 0 (black dots), on day 21 (open circles), and one year after the vaccination (grey triangles) and analyzed by HI test with viruses detected in Finland during influenza seasons 2009–10 (A) and 2010–11 (B). Geometric mean titers (+/− 95% CI) for each virus were calculated. The significance of differences between the egg-grown vaccine virus A/California/07/2009 (Cal E2)-specific antibody responses and multiple Finnish pandemic virus isolates were calculated using Student's t-test, * p<0.01, ** p<0.001, *** p<0.0001.

The antibody titers against different influenza A(H1N1)2009 viruses differ notably. In order to analyze more carefully how the sera from the different donor groups recognize influenza A(H1N1)2009 viruses, we divided the analyzed serum materials in three groups: military conscripts with natural infection-induced immunity [1] (Figure 4A), elderly individuals with cross-reactive immunity against the pandemic virus [4] (Figure 4B), and individuals immunized with the pandemic influenza A(H1N1)2009 vaccine (Figure 5). Among military conscripts who had suffered a natural influenza A(H1N1)2009 virus infection, the geometric mean antibody titers against different viruses ranged between 20 and 105 (Figure 4A). The highest antibody titers (GMT 105) were measured against A/Finland/619/2009, which was the virus that was circulating in the Dragsvik garrison during the outbreak in September 2009. The GMT against this virus formed the baseline to which the responses against the other viruses were compared. The other virus from the same group (II/2009–10) A/Finland/634/2009 as well as A/California/07/2009 (egg-propagated), A/Finland/20/2010 and A/Finland/22/2010 showed similar GMTs in HI titrations. The antibody titers against the other viruses included in these experiments differed statistically significantly or highly significantly from the A/Finland/619/2009 virus-specific responses. Patient anti-sera showed very poor recognition of A/Finland/694/2009 virus (I/2009–10) (Figure 4A). A/Finland/698/2009 (III/2009–10) and A/Finland/618/2009 (IV/2009–10) also gave significantly lower antibody titers as compared to those seen with A/Finland/619/2009 virus. The decrease in antibody levels against A/Finland/694/2009, A/Finland/698/2009, and A/Finland/618/2009 viruses, which exhibited mutations in different antigenic epitopes (N125D and N156K, S74N, or S162R, respectively; Figure 2 and Figure S2) showed that even one or two amino acid change may significantly alter HI titers and also have an effect on seroprotection rates. The seroprotection rates (HI titer ≥40) were 56% lower for A/Finland/694/2009, 24% for A/Finland/698/2009, and 17% for A/Finland/618/2009 compared to that of A/Finland/619/2009 virus (data not shown).

### The elderly have broad-spectrum low-level cross-reactive antibodies against pandemic influenza viruses

We have previously reported that older Finns had cross-reactive antibodies against the pandemic influenza A(H1N1)2009 virus providing potential protection against the infection [4]. We tested a set of serum samples collected in 2005 from individuals whose age was at that time 80 years or over. Among these elderly individuals the antibody titers against the current pandemic viruses were generally low (range of GMTs 10–20). In addition, contrary to patient sera where the antibodies failed to recognize some of the tested viruses, in the elderly none of the tested viruses, except the vaccine strains, were distinguishable in HI test (Figure 4B). This may indicate that infections of the elderly by the Spanish influenza and/or its immediate descendant viruses were able to induce quite broad-spectrum cross-reactive humoral immunity against the pandemic influenza A(H1N1)2009 virus. The significance of the differences was not calculated since none of the studied viruses can be set as a baseline. On the other hand, as compared to the A/California/07/2009 vaccine virus the antibody titers against all the Finnish strains were higher.

### N125D and N156K mutations in Sa epitope of HA compromise the antibody recognition induced by vaccination with pandemic influenza A(H1N1)2009 vaccine

Natural infection with influenza A(H1N1)2009 virus (conscripts) or past infection with Spanish influenza virus (elderly) seemed to raise antibodies that recognized most of the viruses that circulated in Finland during the 2009–2010 and 2010–2011 epidemic seasons. However, viruses which had mutations in the Sa epitope of HA (Figure 2) were more poorly recognized by the patient sera. Next we analyzed how well antibodies, induced by vaccination with the pandemic influenza A(H1N1)2009 vaccine, recognized these viruses and whether the viruses with mutations in antigenic sites can escape from vaccine-induced immunity. Analysis of serum specimens from twenty vaccinated healthy adults showed that 21 days after vaccination anti-influenza antibodies showed a five to fifteen-fold increase in geometric mean titers against all viruses, except that of A/Finland/694/2009 with only less than a three-fold increase (Figure 5A). One year after the vaccination, the mean antibody levels against most studied viruses had clearly decreased but they were still three- to six- folds higher as compared to prevaccination antibody levels. At 12 months post-vaccination A/Finland/694/2009 virus-specific antibody levels were less than two-fold higher over the prevaccination levels. In addition to A/Finland/694/2009, also other viruses (698/2009, 618/2009, 19/2010 and 22/2010) with one or more mutations in the Sa or Cb regions showed significantly lower mean antibody levels as compared to homologous A/California/07/2009 vaccine virus suggesting that minor changes in the surface structure of the HA molecule may affect its immunogenic properties. Interestingly, however, also A/Finland/554/2009 virus, which is almost identical to A/California/07/2009, showed significantly lower antibody levels in the HI test.

### Multiple immunizations of rabbits and guinea-pigs with pandemic influenza A(H1N1)2009 vaccine induced high antibody titers but failed to broaden the initial cross-strain immunity

**Figure 6:**
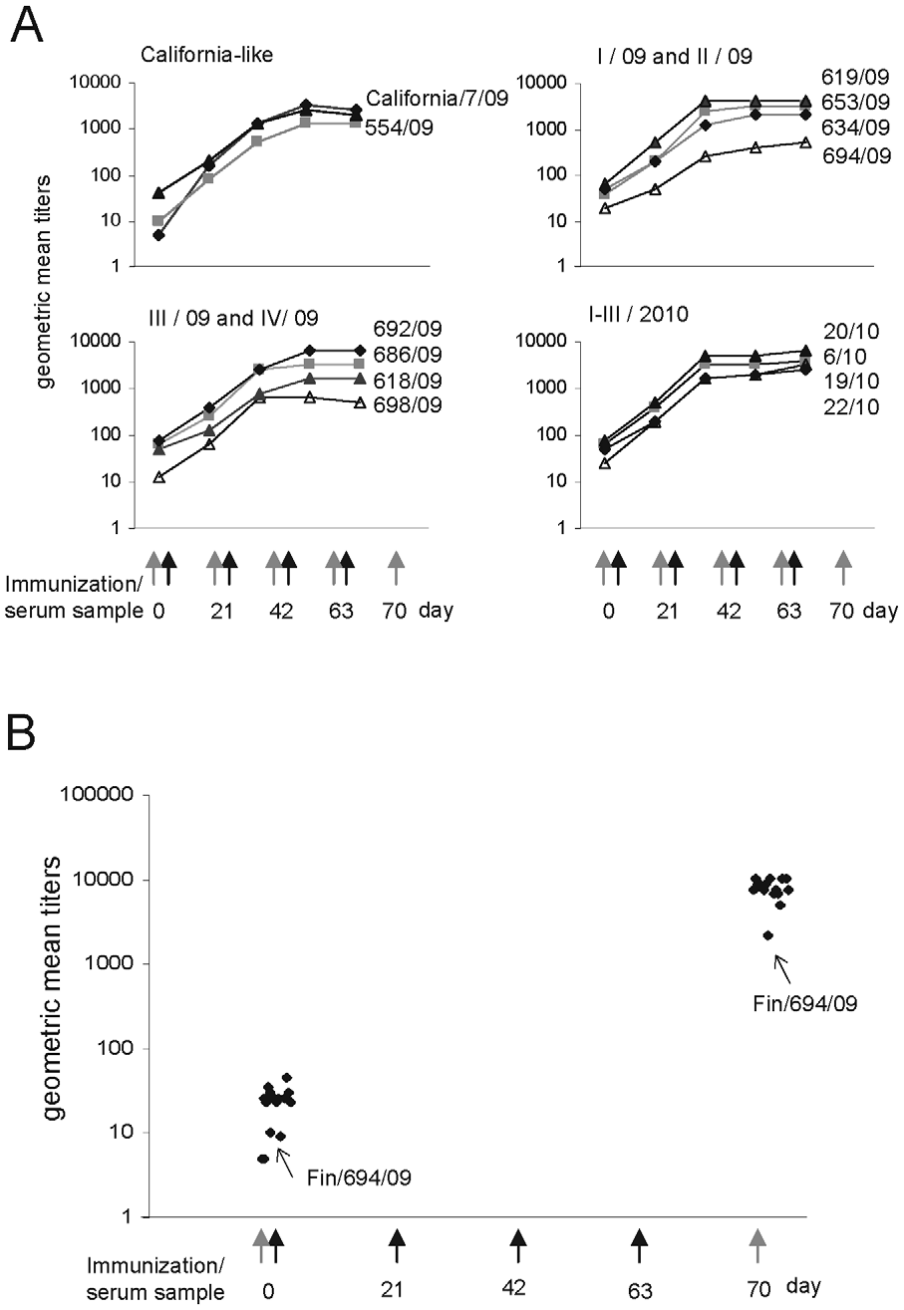
Geometric mean titers of anti-A(H1N1)2009 virus antibodies receiving multiple doses of pandemic influenza A(H1N1) vaccine in rabbits and guinea pigs. Rabbits (A) and guinea pigs (B) were immunized four times at three weeks intervals with pandemic influenza A(H1N1) vaccine and the serum samples were collected on day 0 (rabbits and guinea pigs), before each immunization (rabbits), and one week after the last immunization (rabbits and guinea pigs). The immunizations (black) and sample collections (grey) are marked with arrows. The serum samples were analyzed by HI test and geometric mean titers for each virus were calculated. The means represent the geometric means of three rabbit and five guinea pig anti-sera.

The vaccination of humans against the pandemic influenza A(H1N1)2009 virus with the pandemic influenza A(H1N1)2009 vaccine is usually comprised by one vaccine dose. To analyze whether multiple immunizations would dramatically enhance vaccine induced antibody levels and cross-reactivity against different epidemic A(H1N1)2009 virus strains, we immunized rabbits and guinea pigs up to four times with pandemic influenza A(H1N1)2009 vaccine (Pandemrix, GlaxoSmithKline). Analysis of the mean antibody responses in rabbits (Figure 6A) indicates that the vaccine induces very high antibody levels but for most of the viruses the mean antibody levels reach their maximal or near-maximal levels already after two immunizations. Additional immunizations did not enhance the cross-reactivity of the antibodies against different A(H1N1)2009 strains, since the relative antibody ratios in comparison to vaccine virus were not increased during repeated immunizations (Figure 6A and data not shown). The lowest cross-reactive antibody levels in rabbit anti-sera were the same as compared to human serum specimens and included viruses A/Finland/554/2009, A/Finland/694/2009, A/Finland/618/2009, A/Finland/698/2009, A/Finland/19/2010 and A/Finland/22/2010 (Figures 5 and 6A) further emphasizing true antigenic differences in these viruses as compared to the A/California/07/2009 vaccine virus.

Serum specimens from guinea pigs were collected only at day 0 before the first immunization and one week after the last vaccination. Mean antibody levels increased to very high levels after four doses of vaccine and antibody titers against most viruses were at a comparable level. The only exception was again the A/Finland/694/2009 virus, against which the antibodies remained at much lower level as compared to other viruses (Figure 6B). This suggests that even multiple doses of the vaccine failed to induce highly cross-reactive antibodies against this virus which carries two mutations in antigenic site Sa (Figure 2 and Figure S2).

## Discussion

Genetic characterization of influenza A(H1N1)2009 viruses revealed that virus strains detected in Finland during the 2009–2010 epidemic season fall into four clusters [2] and the most recent 2010–2011 seasonal viruses include three clusters. The different clusters from the epidemic season 2009–2010 and 2010–2011 were distributed as follows I/2009–10 15%, II/2009–10 12%, III/2009–10 11%, IV/2009–10 22%, I/2010–11 22%, II/2010–11 57%, and III 2010–11 20%, respectively. The 2010–2011 Finnish epidemic viruses resemble those that have been found in the Southern hemisphere, Central and Far East Asia, North America, and in Europe. Thus the viruses used in the present study are well representative of globally circulating pandemic A(H1N1)2009 viruses. Comparison of the amino acid sequences of the HA proteins of Finnish A(H1N1)2009 viruses to the A/California/07/2009 vaccine virus HA showed that these viruses have three to eight amino acid changes, of which two to five amino acid changes are located in the most variable parts of the HA molecule, i.e. HA1 region. Molecular characterization of influenza A(H1N1)2009 viruses indicate quite typical (1–1.5% of amino acids per year) rates of evolution and antigenic drift during the past and most recent epidemic seasons [2], [11], [16], [19]. Although the genetic heterogeneity observed among influenza A(H1N1)2009 viruses has been wide, antigenically the viruses have been closely related to the vaccine virus A/California/07/2009. Therefore, the initial pandemic A/California/07/2009 virus remained the WHO recommended vaccine candidate for the 2011–2012 vaccine to be used in the northern hemisphere. This virus is still assumed to give reasonable protection against infection with globally circulating viruses. However, even few amino acid changes in antigenically important sites of the HA, as demonstrated in the present study, may render the vaccine virus a poor match to the circulating virus strains.

The HA protein on the surface of influenza virions is one of the major target molecules for host antibody responses. Therefore, amino acid changes occurring either in the antigenic sites or on the surface of the HA molecule may have an important effect on antibody recognition. Five classical antigenic sites (Sa, Sb, Ca1, Ca2, and Cb) have been described in the HA of influenza A/H1N1 virus [20], [21]. These antigenic sites are located at the globular head of the HA (Figures 1, 2, and S2) although recent studies have described antigenically important determinants also in the stem region of HA [22]–[24]. At present, there is no experimental proof that the antigenic sites found in the HA molecule of classical seasonal H1N1 HA molecules are equally important for the immunity against recent pandemic A(H1N1)2009 viruses. However, our results showed that only one amino acid change in the antigenic site Sa (N125D) compromised antibody recognition, which could be seen as reduced antibody titers in the HI test. This amino acid change is characteristic of group II/2010–11 viruses, which represents the majority of Finnish viruses (57%) from the epidemic season 2010–11. If there was another amino acid change (N156K) in the Sa epitope, as was the case in the A/Finland/694/2009 virus (Figure 2 and Figure S2), the antibody titers were even lower. From all the analyzed viruses (222 strains; data not shown) A/Finland/694/2009 represent the only virus (0.5%) with this double amino acid change. This decrease in antibody titers was seen both in individuals who experienced a natural influenza A(H1N1)2009 infection or who were vaccinated with the pandemic influenza A(H1N1)2009 vaccine. Interestingly, elderly individuals, who were likely infected with the 1918 Spanish influenza and/or its descendant viruses in the early decades of the 20^th^ century, seemed to have low level cross-reactive antibodies against the pandemic A(H1N1)2009 viruses. This cross-reactivity was not sensitive to minor amino acid changes in specific antigenic epitopes, since sera collected from these pre-immune elderly individuals recognized different types of viruses almost equally well.

One of the first outbreaks of influenza A(H1N1)2009 in Finland occurred in the Dragsvik garrison in Southern Finland during September 2009. This outbreak led to the infection of more than 50% of the conscripts as judged by the presence of anti-influenza A(H1N1)2009 virus antibodies after the outbreak [1]. It was very likely that only one virus (A/Finland/619/2009 strain) caused this early outbreak and thus the antibody responses induced by the epidemic virus lead to good recognition of other early pandemic influenza A(H1N1)2009 viruses, such as the A/California/07/2009 vaccine virus and the May 2009 isolate, A/Finland/554/2009. Viruses isolated later during the epidemic, such as the strains A/Finland/694/2009 (changes at positions N125D, N156K and S203T), A/Finland/618/2009 (changes at positions S162R and S203T) and A/Finland/698/2009 (changes at positions S74N and S203T), which showed dual or triple mutations in antigenic sites (Figure 2 and S2) showed impaired recognition by patient convalescent sera. This suggests that relatively strong immune response induced by natural infection might be evaded by only a few mutations in critical antigenic regions. The same effect was also seen among vaccinated individuals, since antibody responses against the above described viruses (694, 618 and 698) were significantly reduced as compared to the homologous vaccine virus strain (Figure 5A). In addition, post-vaccination anti sera showed reduced recognition of epidemic season 2010–2011 viruses A/Finland/19/2010 and A/Finland/22/2010, which showed point mutations in antigenic sites Sa (N125D) and Ca1 (S203T). This further suggests that the specificity of humoral immune responses induced by natural infection and vaccination are alike. In addition, the mean HI titers seen among the vaccinees and those individuals who had undergone a natural infection are comparable (Figure 4A vs. Figure 5) thus indicating that the pandemic influenza A(H1N1)2009 vaccine (Pandemrix) is capable of inducing very good humoral immunity. It is also noteworthy that portion of antibodies that neutralize or in other ways inhibit virus replication may not be detected in HI test. The presence of such antibodies and the possible alterations in antibody recognition of the viruses under discussion would be interesting to analyze in further studies.

An interesting observation in the present study was that three out of four 2010–2011 season viruses had an E374K mutation in the HA2 region (A/Finland/19/2010, A/Finland/20/10, and A/Finland/22/10). Viruses with the same mutation predominated in Oceania during local winter 2010, e.g. the southern hemisphere influenza season 2010 [14]. Barr and coworkers also reported that viruses carrying the N125D and E374K mutations were found in vaccine breakthrough cases in teenagers and adults, who had been vaccinated with the monovalent pandemic influenza A(H1N1)2009 vaccine and the same mutant viruses were also found in a number of fatal cases. However, the two Finnish strains (A/Finland/19/2010 and A/Finland/22/2010) with this dual mutation, representing the epidemic season 2010–2011 group II/10 viruses, were isolated from unvaccinated individuals.

An important question is whether antibody levels and the broadness of cross-reactivity against different virus strains can be stimulated by repeated immunizations with pandemic influenza A(H1N1)2009 vaccine. Since the Finnish population received only one dose of the pandemic vaccine before or during the pandemic we used experimental animals to study the effect of repeated immunizations on antibody responses and cross-reactivity. In rabbits, it was found that already two doses of the pandemic influenza A(H1N1)2009 vaccine were sufficient to induce maximal or near-maximal antibody responses against different epidemic virus strains. Interestingly, repeated immunizations were not able to expand the cross-reactivity against different viruses since the antibody ratios against different virus isolates vs. the vaccine virus strain remained rather constant (Figure 6A). This may indicate that the initial specificity of the antibody response is determined at early times of immunization and repeated immunizations with the same vaccine, such as the Pandemrix vaccine, does not lead to broadening of the specificity of the antibody response. These findings may indicate that also in humans, in order to induce better antibody cross-reactivity, the composition of the vaccine has to be changed rather than giving repeated immunizations with the same vaccine.

The present study demonstrates that circulating influenza A(H1N1)2009 viruses are genetically highly diverse and at present a specific globally circulating virus strain has not been observed. Genetic characterization of the Finnish circulating viruses, which originated from different parts of the world, indicates that the evolutionary speed of the virus is quite typical for influenza A viruses. However, there appears to be a tendency that many viruses have mutations in antigenically important epitopes of the HA molecule and these mutations may have quite dramatic effects on antibody recognition both among vaccinated individuals and those that had experienced a natural infection. Thus, continuous genetic and antigenic surveillance of circulating influenza viruses as well as rapid and open sharing of information is of utmost importance for WHO to make the best possible recommendation for the vaccine viruses. In addition, it is important to rapidly identify virus strains with elevated virulence or with the ability to escape vaccine- or natural infection-induced immunity.

## Materials and Methods

### Viruses

All viruses for serological analysis were selected based on their HA gene nucleotide sequence. We have previously reported that based on the HA gene, influenza A(H1N1)2009 viruses circulating in Finland during 2009–2010 season clustered in four genetic groups [2]. Two representatives of each of these four groups were selected for immunological analyses: A/Finland/618/2009, A/Finland/619/2009, A/Finland/634/2009, A/Finland/653/2009, A/Finland/686/2009 A/Finland/692/2009 A/Finland/694/2009, and A/Finland/698/2009. One additional virus, A/Finland/554/2009 [17], which was one of the first influenza A(H1N1)2009 viruses isolated in Finland in 2009 and is very closely related to the A/California/07/2009 vaccine virus, was also included in the analyses. Four more recent viruses, A/Finland/6/2010, A/Finland/19/2010, A/Finland/20/2010 and A/Finland/22/2010, were chosen for analyses to represent the epidemic season 2010–2011. All viruses were propagated in MDCK cells and stored in aliquots at −80°C. For comparison A/California/07/2009 was also grown in embryonated hens' eggs since the pandemic influenza A(H1N1) vaccine was prepared from an egg-grown virus.

### Serum specimens

In order to get a broader view of the antigenic characteristics of the selected viruses, we chose three different sets of serum specimens for serological analysis. The first set of sera was from 20 healthy adults who received pandemic influenza A(H1N1) vaccine *Pandemrix* in November 2009. *Pandemrix* is a monovalent split-virion vaccine generated from the A/California/07/2009-like strain (NYMC X-179A; New York Medical College) by GlaxoSmithKline (GSK) Biologicals [25]. A single dose of this vaccine contains 3.75 µg of HA and AS03 adjuvant. Serum samples from 20 volunteers (median age 42; range 27 to 62 years) were collected prior to vaccination on day 0, day 21, and one year after the vaccination. The second set of sera was collected from 50 unvaccinated military conscripts (median age 21; range 20–28) in the Dragsvik garrison (Raasepori), which is located in Southern Finland. An outbreak of influenza A(H1N1)2009 virus occurred in this garrison during September 2009 and 50% of the conscripts were infected (confirmed by PCR) with influenza A(H1N1)2009 [1]. For the present study we chose 40 A(H1N1)2009 virus seropositive and 10 seronegative randomly selected serum specimens. The third set of serum samples was obtained from the virus diagnostic unit at the Central Laboratory Services of the Helsinki University Hospital (HUSLAB). These sera had been collected prior to influenza A(H1N1)2009 pandemic in 2004–2005 and the specimens have been randomly selected from all sera sent to HUSLAB from different parts of the country. The samples were anonymous, only the age of the donor and sample collection date was known. For the present study, 50 serum samples from elderly individuals representing the age group of 80 years or older were chosen, since our previous observations indicated that these individuals have cross-reactive antibodies against influenza A(H1N1)2009 virus [4].

The collection of serum samples for the present study was approved by the Ethical Committee of the Helsinki-Uusimaa Health District (Permissions 382/E5/07 §48/2008 and §289/2010 and 199/13/03/00/2009 §164). All individuals participating in the studies (vaccinated and conscripts) gave written informed consent before enrolment in the study.

### Sequence and phylogenetic analysis of HA gene

The nucleotide sequences of the influenza A(H1N1)2009 viruses for this study (35 Finnish strains from the epidemic season 2010–2011) were sequenced from original clinical samples. RNA extraction, cDNA synthesis, DNA amplification and sequencing were performed as previously described [2]. The nucleotide sequences of the complete HA genes were assembled using Sequencer version 4.7 (Gene Codes Corporation, Ann Arbor, USA). Mega software version 4 [26] (Molecular Evolutionary Genetics Analysis) was used in nucleotide and amino acid sequence comparison and for the construction of the phylogenetic tree. The Neighbor-joining method [27] with the maximum composite likelihood model [28] was used to generate the phylogenetic tree. Bootstrapping was performed with 1000 replicates [29]. In the present study all HA amino acid residues are numbered without the signal peptide sequence.

GenBank accession numbers of the HA sequences of the Finnish virus strains from the epidemic season 2009–2010 are GQ283488, GU292341, HQ228034, HQ228037, HQ228039-40, HQ228042, HQ228053-4, HQ228057, HQ228059-60, HQ228067-8, HQ228074, HQ228076, HQ228078-80, HQ228083-4, HQ228086, HQ228090-1, HQ228093-6, HQ228098, HQ228103-4, HQ228107, HQ228109, HQ228111, HQ228113-4, HQ228118, HQ228120-1, HQ228125, HQ228127, HQ228129, HQ228131, HQ228133-4, HQ228137-8, HQ228140, HQ228142, and HQ228144-7 and from the season 2010–2011 they are JN601076-JN601110. The GenBank accession number for the vaccine strain, A/California/07/2009 is FJ966974. Supplemental sequences for the phylogenetic tree were obtained from GISAID EpiFlu™Database. Identification codes of supplemental sequences are listed in Figure S3. The GenBank accession number for A/South Carolina/1/18 is AF117241, for A/Puerto Rico/8/1934 CY033577 and for A/New Jersey/8/1976 CY039991. The clinical samples for isolation of viruses included in this study have been collected for routine viral diagnostic purposes. Based on national laws ethical permission are not required for specific microbiological diagnostics and further characterization of detected viruses.

### Structural analysis of amino acid differences in the HA molecule

The three-dimensional structure of the HA molecule of influenza A(H1N1)2009 virus, A/California/04/2009 (RCSB Protein Bank accession number 3LZG) was used to locate amino acid differences between the Finnish A(H1N1)2009 viruses (seasons 2009–2010 and 2010–2011) and the A/California/07/2009 vaccine virus. The molecular models were constructed using RasMol Molecular Graphics software version 2.7.3 [30].

### Immunizations of rabbits and guinea pigs

In order to study the immunogenicity and the ability of the pandemic influenza A(H1N1)2009 vaccine to induce cross-reactive antibody responses in animals we immunized five guinea pigs and three rabbits four times in three weeks intervals. Each animal received one human dose (3,75 µg HA in AS03 adjuvant) of vaccine with each immunization. Serum samples from guinea pigs were collected before the first immunization (day 0) and one week after the last immunization (day 70). From the rabbits serum samples were collected on day 0, before each immunization on days 21, 42, and 63, and one week after the last immunization on day 70. Immunizations of the animals and the collection of serum samples were approved by the Ethical Committee of National Institute for Health and Welfare (permission KTL 2008-02). Serum samples were stored at −20°C and analyzed for influenza A virus specific antibodies by the HI test.

### Serologic assays

All serum specimens were assayed by the HI test against nine influenza A(H1N1)2009 strains isolated in Finland during the 2009–2010 epidemic season, four Finnish influenza A(H1N1)2009 strains from season 2010–2011, and A/California/07/2009 vaccine strain. The HI tests were performed in accordance with WHO guidelines with established procedures [31], [32] using turkey erythrocytes. In brief, serum samples were pretreated with receptor destroying enzyme (RDE) from Vibrio cholerae filtrate (Denka Seiken, Tokyo, Japan) at +37°C for 18 h and then with 100% turkey erythrocytes at +4°C for one hour to remove non-specific inhibitors and agglutinins. Serial 1∶2 serum dilutions starting from 1∶10 initial dilution were made, live viral antigen (4 HA units) was added and incubated for one hour at ambient temperature. Turkey red blood cells (0.5%) were added to antigen-serum solutions and incubated at room temperature for 30 minutes to determine the HI titers. Serum specimens with HI titers <10 were assigned a titer value of 5 for statistical analyses.

### Statistical analysis

Antibody titers obtained by the HI test were analyzed statistically by calculating geometric mean titers with 95% confidence intervals for each virus. Significance of the differences between mean titers for each virus compared to the vaccine strain was calculated using Student's t-test (paired, two-tailed) and the significance level was adjusted to p<0.01. The correlation coefficient and the significance of the correlation coefficient of the antibody titers for each virus were calculated against the A/California/07/2009 egg-grown virus.

## Supporting Information

Figure S1 **Amino acid sequence alignment of mature HA protein of the Finnish influenza A(H1N1)2009 viruses and some representative A/H1N1 viruses with the vaccine strain A/California/07/2009.** Antigenic sites are marked as Sa in red, Sb in blue, Ca1 in darker green, Ca2 in lighter green and Cb in orange. The unchanged amino acids (compared to A/California/07/2009) are marked with dots and amino acid changes with corresponding letter. The GenBank accession numbers of the HA sequences of Finnish strains are GQ283488, HQ228067-8, HQ228083, HQ228096, HQ228125, HQ228131, HQ228133, HQ228137, JN601076, JN601088-9, and JN601091. The accession number for A/California/07/2009 vaccine virus strain is FJ966974, for A/South Carolina/1/18 is AF117241, A/Puerto Rico/8/1934 is CY033577 and A/New Jersey/8/1976 is CY039991.
(TIF)

Figure S2 **Schematic representation of amino acid differences in the HA between Finnish influenza A(H1N1)2009 viruses and the vaccine virus, A/California/07/2009.** In the middle of the figure a top view of trimeric structure of HA molecule of influenza A(H1N1(2009) (A/California/04/2009; RCSB Protein Bank accession number 3LZG) with previously identified H1 protein-specific antigenic sites (Sa in red, Sb in blue, Ca1 in darker green, Ca2 in lighter green and Cb in orange) of influenza A(H1N1) viruses and with the receptor binding pocket (RBP, purple) is shown. Different monomers are shown in various shades of grey color. The amino acid changes of Finnish A(H1N1)2009 viruses compared to A/California/07/2009, the vaccine strain, are illustrated in the trimeric HA structure. Amino acid changes in the antigenic sites are colored as in A/California/04/2009 virus HA molecule. Amino acid changes outside the expected antigenic sites are shown in yellow. Changes are illustrated by amino acid residue number and with serial number of virus where the respective amino acid change has been observed.
(TIF)

Figure S3 **Table of identification codes for the supplemental sequences for phylogenetic tree obtained from GISAID EpiFlu™Database.**
(TIF)
