# Supplementary figures and images for: Minor Changes in the Hemagglutinin of Influenza A(H1N1)2009 Virus Alter Its Antigenic Properties
Source: PLoS One. 2011 Oct 11;6(10):e25848. doi: 10.1371/journal.pone.0025848 (PMC3191144; doi:10.1371/journal.pone.0025848)

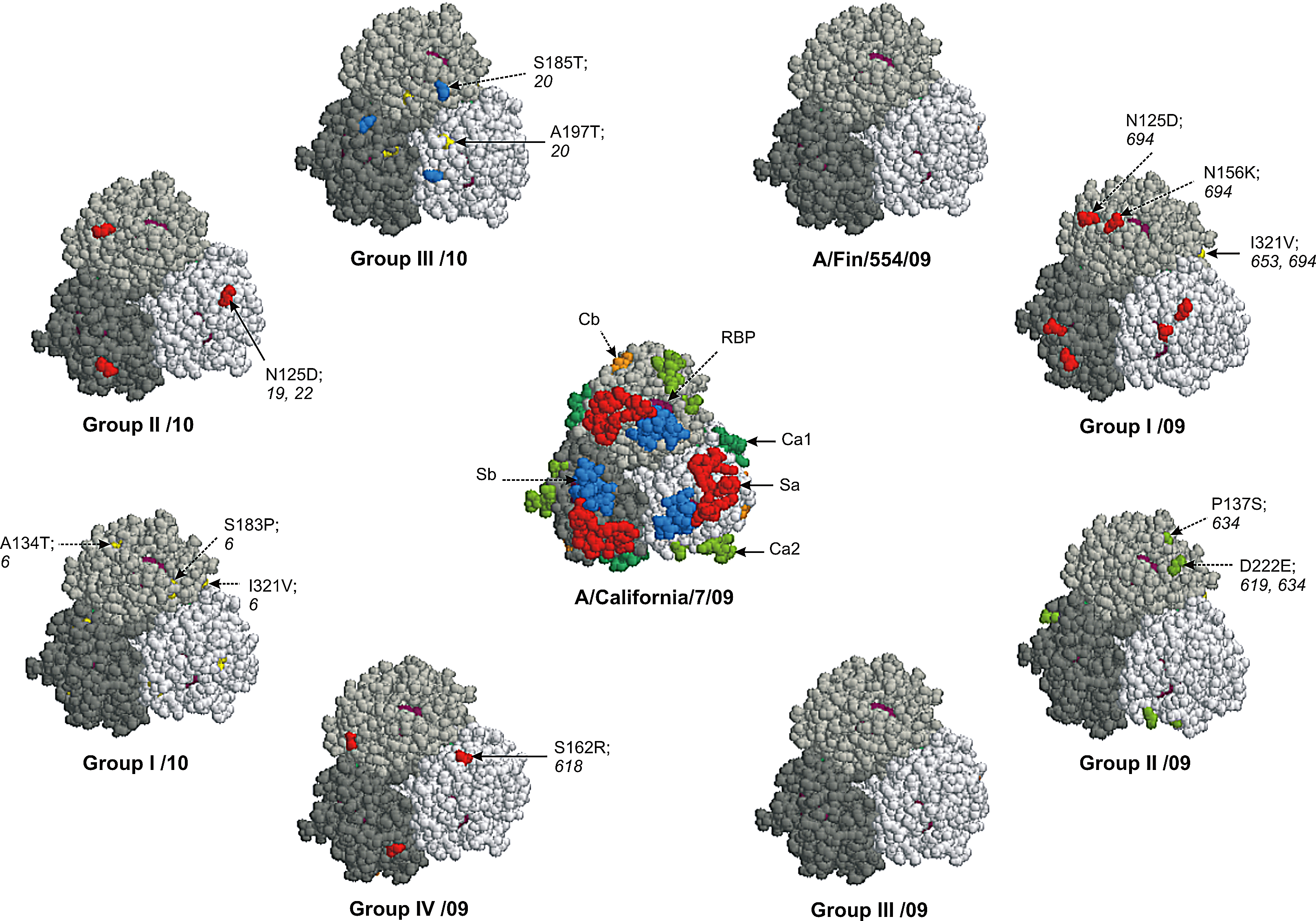

Supplement: Figure S2 — Schematic representation of amino acid differences in the HA between Finnish influenza A(H1N1)2009 viruses and the vaccine virus, A/California/07/2009. In the middle of the figure a top view of trimeric structure of HA molecule of influenza A(H1N1(2009) (A/California/04/2009; RCSB Protein Bank accession number 3LZG) with previously identified H1 protein-specific antigenic sites (Sa in red, Sb in blue, Ca1 in darker green, Ca2 in lighter green and Cb in orange) of influenza A(H1N1) viruses and with the receptor binding pocket (RBP, purple) is shown. Different monomers are shown in various shades of grey color. The amino acid changes of Finnish A(H1N1)2009 viruses compared to A/California/07/2009, the vaccine strain, are illustrated in the trimeric HA structure. Amino acid changes in the antigenic sites are colored as in A/California/04/2009 virus HA molecule. Amino acid changes outside the expected antigenic sites are shown in yellow. Changes are illustrated by amino acid residue number and with serial number of virus where the respective amino acid change has been observed. (TIF) [file pone.0025848.s002.tif]
